# Unlocking the serine mischarging paradox and inhibiting lactyltransferase activity of AlaRS by a single-point mutation

**DOI:** 10.1093/nar/gkaf462

**Published:** 2025-06-06

**Authors:** Wooyoung Park, Se-Young Son, Joonyeop Yi, Seungwoo Cha, Hankyeol Moon, Minyoung Kim, Sangho Ji, Wookyung Yu, Changmin Sung, Sun-Shin Cha, Ji-Sook Hahn

**Affiliations:** Department of Chemical and Biological Engineering, Institute of Chemical Processes, Seoul National University, Seoul 08826, Republic of Korea; Department of Chemistry & Nanoscience, Ewha Womans University, Seoul 03760, Republic of Korea; Doping Control Center, Korea Institute of Science and Technology, Seoul 02792, Republic of Korea; Interdisciplinary Program for Biochemical Engineering and Biotechnology, Seoul National University, Seoul 08826, Republic of Korea; Department of Chemical and Biological Engineering, Institute of Chemical Processes, Seoul National University, Seoul 08826, Republic of Korea; Department of Chemical and Biological Engineering, Institute of Chemical Processes, Seoul National University, Seoul 08826, Republic of Korea; Doping Control Center, Korea Institute of Science and Technology, Seoul 02792, Republic of Korea; Interdisciplinary Program for Biochemical Engineering and Biotechnology, Seoul National University, Seoul 08826, Republic of Korea; Department of Brain Sciences, DGIST, Daegu 42988, Republic of Korea; Department of Brain Sciences, DGIST, Daegu 42988, Republic of Korea; Doping Control Center, Korea Institute of Science and Technology, Seoul 02792, Republic of Korea; Department of Chemistry & Nanoscience, Ewha Womans University, Seoul 03760, Republic of Korea; Graduate Program in Innovative Biomaterials Convergence, Ewha Womans University, Seoul 03760, Republic of Korea; Department of Chemical and Biological Engineering, Institute of Chemical Processes, Seoul National University, Seoul 08826, Republic of Korea; Interdisciplinary Program for Biochemical Engineering and Biotechnology, Seoul National University, Seoul 08826, Republic of Korea

## Abstract

Aminoacyl-tRNA synthetases are critical for accurate genetic translation, attaching amino acids to their corresponding transfer RNA molecules. Alanyl-tRNA synthetase (AlaRS) often misactivates Ser or Gly instead of Ala, which is detrimental unless corrected by its editing functions. The paradox of misactivating larger Ser by AlaRS was considered inevitable due to its inherent design, sharing an essential acidic residue to accommodate the activated adenylated intermediates from both cognate and non-cognate amino acids. Here we show a groundbreaking discovery where a single-point mutation, L219M, in AlaRS from *Methylomonas* sp. DH-1, effectively eliminates Ser misactivation. Structural analysis of the pre-activation state unveiled that the flexibility of Val204 is the key to preventing Ser binding in AlaRS^L219M^. This research elucidates the amino acid discrimination mechanism in AlaRS, independent of editing domain. Remarkably, the AlaRS^L219M^ mutation was initially identified as a causal mutation enhancing lactate tolerance in a strain developed through adaptive laboratory evolution. We showed that AlaRS^L219M^ also eliminates the enzyme’s inherent lactyltransferase activity, suggesting that the lactate tolerance observed might result from preventing excessive protein lactylation under lactate stress. This opens possibilities for developing high-fidelity and lactylation-deficient AlaRS mutants across various organisms, facilitating studies on their potential benefits in different physiological scenarios.

## Graphical Abstract

**Graphical Abstract:**
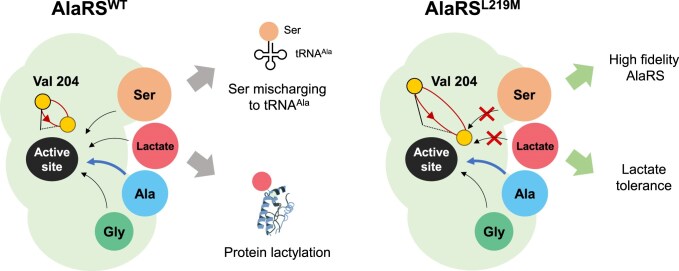

## Introduction

Aminoacyl-tRNA synthetases (AARSs) play a crucial role in the translation of genetic information by attaching specific amino acids to their corresponding transfer RNA (tRNA) molecules [1]. This tRNA aminoacylation reaction occurs in two steps: first, the amino acid is activated with ATP to form an aminoacyl-adenylate, releasing pyrophosphate; second, the activated amino acid is transferred to the 3′ end of its corresponding tRNA, releasing AMP. Most living cells contain a set of 20 AARSs, with each AARS dedicated to a specific amino acid [1].

The accuracy of tRNA aminoacylation is critical for the precise expression of the genetic code. To avoid mischarging, approximately half of all AARSs possess an editing mechanism, which involves both pre-transfer and post-transfer editing steps [2, 3]. Pre-transfer editing encompasses the hydrolysis of the misactivated amino acid prior to its attachment to the tRNA molecule. On the other hand, post-transfer editing is the process of hydrolyzing a mischarged tRNA at the editing site [2, 4, 5]. Alanyl-tRNA synthetase (AlaRS) often misactivates Gly or Ser instead of its cognate amino acid, Ala [6]. The paradox of misactivating Ser, which is larger than Ala, has been elucidated by examining the shared binding pattern among Ala and the non-cognate amino acid moieties of aminoacyl-adenylates after the activation step [7, 8]. The structural study of *Escherichia coli* AlaRS complexed with an intermediate (alanyl-adenylate) or intermediate analogs (Ala-SA, 5′-*O*-(*N*-(l-alanyl)-sulfamoyl) adenosine; Ser-SA; and Gly-SA) revealed that the α-amino groups of their amino acid moieties each engage in interactions with a conserved acidic residue, Asp235, within the binding pocket. This acidic residue also participates in an interaction with the hydroxyl group of seryl moiety and contributes to the tight binding of Ser-SA to the active site. It makes a substantial challenge for AlaRS, as it lacks sufficient accuracy in intermediate discrimination after the activation step [8]. As a result, post-transfer editing becomes crucial for correcting the mis-aminoacylation errors. The editing process is facilitated through the editing domain within AlaRS, as well as AlaXp, a trans-acting factor that exists as an independent protein. AlaXps exhibit homology to the editing domain of AlaRS and are conserved throughout all three domains of life [2, 9–11]. Editing-defective AlaRS has been associated with numerous disease conditions, including cerebellar Purkinje cell loss, ataxia, and cardiomyopathy in mice, perinatal or infantile cardiomyopathy in human, and changes in phenotype such as slow growth in *E. coli* and attenuated heat shock response in yeast [12–17]. These findings indicate that even a slight increase in mischarging can adversely affect cellular function. Toxicity resulting from an editing defect in AlaRS appears to be more problematic with Ser than with Gly [12, 14, 15].

Methane, obtained from natural gas and biogas, is an abundant and inexpensive carbon source. It is also a potent greenhouse gas with a high global warming potential [18]. Therefore, there is a growing interest in utilizing methane as a next-generation feedstock. Methanotrophs, which utilize methane as a carbon and energy source, are promising hosts for the bioconversion of methane into value-added chemicals [19–23]. In a previous study, we engineered a methanotrophic bacterium *Methylomonas* sp. DH-1 to produce lactic acid from methane [19]. The inhibitory effects of lactate pose a challenge in achieving high-level lactate production. Therefore, we improved lactate production by using a lactate-tolerant strain, JHM80, generated through adaptive laboratory evolution (ALE) [19].

In this study, we subjected JHM80 to additional rounds of ALE, resulting in a JHM102 strain with enhanced lactate tolerance. By conducting whole-genome sequencing of JHM102, we identified a mutation, L219M, in AlaRS that is responsible for the enhanced lactate tolerance observed in this strain. Biochemical and structural studies have shown that this mutant enhances fidelity by decreasing the misactivation of Ser. This is the first discovery of an AlaRS mutant capable of distinguishing the incorrect substrate Ser during the activation step without relying on the editing domain. This achievement was previously supposed impossible due to the inherent design of AlaRS.

Interestingly, recent findings reveal that AlaRS also functions as a lactate sensor and lactyltransferase, mediating global Lys lactylation across various species, from prokaryotes to mammals [24–26]. We discovered that the high-fidelity L219M mutation in AlaRS prevents not only Ser activation but also lactate activation, potentially eliminates global lysine lactylation under lactate stress conditions, thereby enhancing lactate tolerance.

## Materials and methods

### Strains and culture conditions

All strains used in this study are listed in Supplementary Table S1. Strains were derived from *Methylomonas* sp. DH-1 (KCTC13004BP) and cultured in nitrate mineral salts (NMS) medium [0.49 g/l MgSO_4_, 1.0 g/l KNO_3_, 0.23 g/l CaCl_2_·2H_2_O, 3.8 mg/l Fe–EDTA (ethylenediaminetetraacetic acid), 0.5 mg/l Na_2_MoO_4_, 10 μM CuSO_4_·5H_2_O, with the addition of 1000× trace element solution, 100× vitamin stock solution, and 100× phosphate stock solution]. Detailed recipes of these solutions are provided in Supplementary Table S2. Strains were grown in 3 ml NMS medium supplemented with 20% (v/v) methane in a 30-ml serum bottle capped with a butyl rubber stopper or in 12.5 ml NMS medium in a 125 ml baffled flask sealed with rubber type screw cap at 30°C with shaking at 170 rpm [19].

### Genetic manipulation of *Methylomonas* sp. DH-1

Plasmids and primers used in this study are listed in Supplementary Tables S3 and S4. Plasmids for introducing mutations to AlaRS of DH-1 or JHM80 were generated based on the pIns plasmid [19]. DNA cassettes containing [*alaRS*–*aspK*] and [*alaRS^L219M^*–*aspK*] were amplified by PCR from the genomic DNA of *Methylomonas* sp. DH-1 wild type and strain JHM102, respectively. Each cassette included the region from 1 kb upstream of the *alaRS* gene to 1 kb downstream of *aspK* (AYM39_07160). These fragments were sequentially cloned into the NotI/BcuI and ApaI/SacI sites of pIns vector, resulting in the constructs pIns-alaRS and pIns-alaRS^L219M^. The plasmids pIns-alaRS^L219F^ and pIns-alaRS^L219A^ were generated from pIns-alaRS using PCR-based site-directed mutagenesis. Gene substitutions in DH-1 and JHM80 strains were performed as previously described [19].

### Adaptive laboratory evolution

To improve the lactate tolerance of JHM80, cells were adapted to lactate by gradually increasing the concentrations of lactic acid in NMS media from 8 to 10.2 g/l during growth. The pH of the NMS medium containing lactic acid was adjusted to 6.8 with NaOH.

### Whole-genome sequencing

Genomic DNAs of JHM80 and JHM102 were isolated using a bacteria genomic DNA extraction kit (iNtRON Biotechnology, Republic of Korea). DNA libraries were generated by using a TruSeq Nano DNA LT Kit (Illumina, USA) and sequenced using PE 2 × 300-MiSeq (Illumina, USA). Mutated DNA sequences in JHM80 and JHM102 were analyzed as previously described [27].

### Cloning, expression, and purification for aminoacylation assay

Plasmids for the expression of *alaRS* in *E. coli* were constructed in pET-28b(+) vector (Novagen, USA). The open reading frame (ORF) of *alaRS* and its variants were amplified by PCR from pIns-alaRS, pIns-alaRS^L219M^, pIns-alaRS^L219F^, or pIns-alaRS^L219A^ using primers containing BamHI*/*NotI sites and ligated into pET-28b(+). pET-28b(+)-AlaRS^V204A L219M^ and pET-28b(+)-AlaRS^V204L L219M^ were generated from pET-28b(+)-AlaRS^L219M^, and pET-28b(+)-AlaRS^V204A^ and pET-28b(+)-AlaRS^V204L^ were generated from pET-28b(+)-AlaRS by PCR-based mutagenesis. The plasmids were transformed into *E. coli* Rosetta-gami2(DE3)pLysS (Novagen, USA), and the cells were grown at 37°C. One millimolar isopropyl β-d-1-thiogalactopyranoside (IPTG) was added at an OD_600_ of 0.6–0.7 and further grown for 4 h at 30°C. Proteins were purified from cell extracts by using Ni-NTA resin (Thermo Fisher Scientific, USA). The elute was dialyzed against the dialysis buffer [50 mM Tris (pH 7.5), 150 mM NaCl, 15% (v/v) glycerol].

### Aminoacylation assay and lactylation assay

The *in vitro* AlaRS activity assay was performed to measure the amount of reacted amino acids by detecting the pyrophosphate (PPi) released during ATP hydrolysis in the amino acid activation step. tRNA^Ala^ of *Methylomonas* DH-1 was prepared by *in vitro* transcription using TranscriptAid T7 High Yield Transcription Kit (Thermo Fisher Scientific, USA). PCR amplification of tRNA^Ala^ was performed from genomic DNA of DH-1 with forward primer containing T7 promoter sequence. tRNA^Ala^ was extracted with phenol (pH 4.7):chloroform and the aqueous phase containing tRNA^Ala^ was precipitated with ethanol. tRNA^Ala^ was folded before the aminoacylation assay by heating at 70°C for 10 min followed by addition of 10 mM of MgCl_2_ and slow cooling in room temperature for 5 min [28]. The enzyme assay was conducted in an aminoacylation buffer composed of 30 mM HEPES (pH adjusted to pH 7.5 with KOH), 140 mM NaCl, 30 mM KCl, 40 mM MgCl_2_, supplemented with 1 mM dithiothreitol (DTT), 200 μM ATP, 2 U/ml inorganic pyrophosphatase, and 100 μg/ml enzyme. The enzyme concentration corresponded to 1.01 μM for wild-type AlaRS, AlaRS^L219M^, AlaRS^L219F^, AlaRS^L219A^, AlaRS^V204A L219M^, AlaRS^V204L L219M^, AlaRS^V204A^, and AlaRS^V204L^, and 2.06 μM for wild-type AlaRS_429_ and AlaRS_429_^L219M^. Amino acids such as Ala, Ser, Gly, or Pro were added at varying concentrations along with 8 μM tRNA^Ala^; Ala and Ser were also tested in the absence of tRNA. Reactions were carried out in a 96-well plate at 37°C. Aliquots of 10 μl were collected at 0, 5, 10, 15, 20, and 25 min, and immediately quenched with ice-cold 10 mM EDTA to terminate the reaction. Samples were kept at 0°C thereafter. To quantify phosphate released from PPi by the action of inorganic pyrophosphatase, 100 μl of BIOMOL^®^ Green reagent (Enzo Life Sciences, USA) was added to each time-point sample. These samples were then incubated at room temperature for 20 min before the absorbance was measured at 620 nm [28, 29]. The initial reaction velocity was determined by calculating the slope of the substrate conversion over time. For AlaRS^L219F^, AlaRS^L219A^, and AlaRS^V204L L219M^, the PPi release rate was measured at the 15-min mark, as the reaction remained linear for up to 30 min. A plot of reaction velocity versus substrate concentration was generated to illustrate their relationship. Kinetic parameters (*K*_m_ and *k*_cat_) were calculated using the Michaelis–Menten equation, with curve fitting performed using the Curve Fitting Toolbox 3.5.13 in MATLAB R2021a. All assays were conducted in triplicate to ensure reproducibility.

The lactylation assay was carried out using 100 μg/ml of wild-type AlaRS and AlaRS^L219M^ in an aminoacylation buffer containing 1 mM DTT, 200 μM ATP, 2 U/ml inorganic pyrophosphatase, and 10 mM lactate (pH adjusted to 7.5 with NaOH) [24]. Reaction mixtures were incubated at 37°C for 15 min, and then quenched by the addition of ice-cold 10 mM EDTA and kept at 0°C. Subsequently, 100 μl of BIOMOL^®^ Green reagent was added to each sample, followed by a 20-min incubation at room temperature before measuring absorbance at 620 nm.

### Cloning, expression, and purification for crystallography

The *Methylomonas* sp. DH-1 gene encoding residues 1–429 of AlaRS (AlaRS_429_) was synthesized to have the in-frame non-cleavable C-terminal His6-tag (inserted between the XbaI and XhoI sites of the pET28b(+) vector. The L219M mutant of AlaRS (AlaRS_429_^L219M^) was generated by site-directed mutagenesis using the wild-type gene as a template. After gene insertion, expression vectors were transformed into *E. coli* strain BL21 (DE3). The transformants were grown in Luria–Bertani media containing 50 μg/ml kanamycin at 37°C, and 1 mM IPTG was added at an OD_600_ of 0.6–0.7. After 4-h induction at 37°C, the cells were harvested, resuspended in a buffer containing 20 mM Tris (pH 8.0), 500 mM NaCl, and 2 mM β-mercaptoethanol, and disrupted by sonication. The crude lysate was centrifugated at 15 000 × *g* for 40 min. The resulting supernatant was loaded onto a Ni-NTA column (GE Healthcare, USA). The eluted fractions by 250 mM imidazole containing AlaRS were applied to the Q Sepharose^®^ Fast Flow column (GE Healthcare) equilibrated with A buffer [20 mM Tris (pH 8.0) and 2 mM β-mercaptoethanol]. The elution was performed using a linear gradient of 0–1.0 M NaCl in A buffer. The fractions containing AlaRS were finally loaded onto a Superdex^®^ 200 16/600 column (GE Healthcare) equilibrated with a buffer consisting of 20 mM Tris (pH 8.0), 500 mM NaCl, and 2 mM β-mercaptoethanol. The elute from gel filtration was dialyzed and concentrated to ∼17 mg/ml in 5 mM Tris (pH 8.0), 50 mM NaCl, and 2 mM β-mercaptoethanol for crystallization.

### Crystallization, data collection, and structure determination

The microbatch crystallization method was employed to grow crystals under oil [30, 31]. Crystals of AlaRS_429_ were obtained at 22°C by mixing 1 μl of protein solution with an equivalent volume of a mother liquor consisting of 16% polyethylene glycol (PEG) 3350, 2% (v/v) Tacsimate™ (pH 5.0), and 100 mM sodium citrate tribasic dihydrate (pH 5.0). AlaRS_429_^L219M^ was crystallized in the same way with a mother liquor of 16% (w/v) PEG 3350, 50 mM citric acid, and 50 mM bis-tris propane (pH 5.0). When the substrates were soaked into the crystals of AlaRS, the resulting F_o_–F_c_ omit map (green) contoured at 4*σ* at the active site revealed variations depending on the substrate concentration and soaking time. We attempted to collect X-ray diffraction data from as many crystals as possible under various conditions. Ultimately, we successfully obtained two diffraction datasets after soaking times of 6 h (AlaRS_429_) and 10 min (AlaRS_429_^L219M^) at room temperature in the same precipitant solution supplemented with 25 mM ATP and 25 mM Ala. For data collection, crystals were frozen after a brief soak in a cryoprotectant solution containing 20% ethylene glycol (AlaRS_429_) and 20% glycerol (AlaRS_429_^L219M^) in the same precipitant solution. A 1.90-Å resolution dataset of AlaRS_429_ in complex with ATP and Ala and a 2.18-Å resolution dataset of AlaRS_429_^L219M^ in complex with ATP and Ala were collected from a flash-cooled crystal at 100 K at beamline 5C of Pohang Light Source, Republic of Korea (Supplementary Table S5). Both datasets were integrated and scaled with X-ray detector software (XDS). Crystals of both proteins belonged to the space group *P*2_1_ with cell parameters (AlaRS_429_/AlaRS_429_^L219M^) of *a* = 78.23/78.84 Å, *b* = 55.59/56.57 Å, *c* = 125.72/125.63 Å, and *β* = 102.1/102.3° corresponding to two monomers in an asymmetric unit. Molecular replacement was performed with PHASER using the structure of *E. coli* AlaRS (PDB code: 3hy1) as a search model. Solutions from PHASER were manually manipulated by COOT and refined by PHENIX [32, 33]. Several rounds of refinements and manual refitting gave rise to final models of AlaRS_429_ (*R*/*R*_free_ = 0.1983/0.2298) and AlaRS_429_^L219M^ (*R*/*R*_free_ = 0.2013/0.2336) (Supplementary Table S5). The final models of two AlaRS_429_ molecules contain residues 1–425, while those of AlaRS_429_^L219M^ consist of residues 1–70 and 74–425 (chain A) and residues 1–71 and 74–425 (chain B). The Ramachandran plots indicate that 97.40% (AlaRS_429_) and 97.13% (AlaRS_429_^L219M^) of non-glycine residues are in the most favored regions, and all others are in the additionally allowed regions.

## Results

### L219M mutation in AlaRS is responsible for the lactate tolerance in *Methylomonas* sp. DH-1

**Figure 1.**
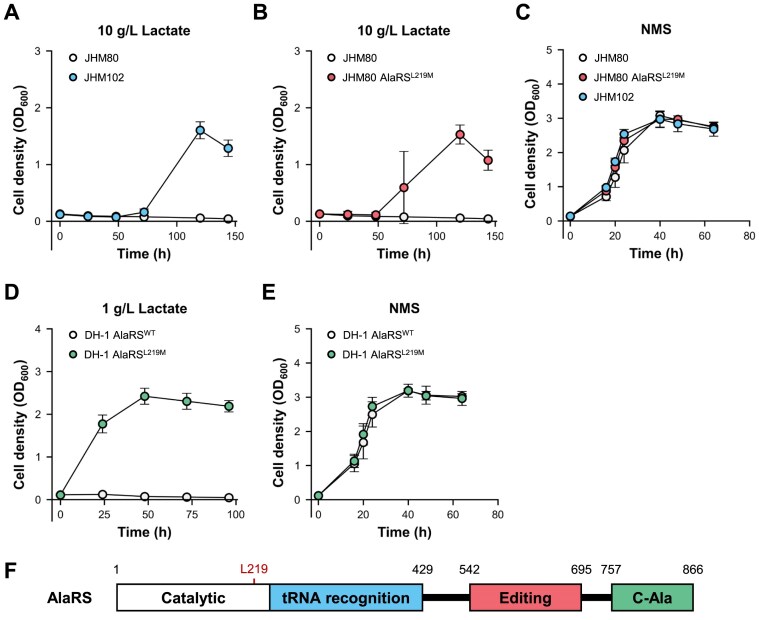
AlaRS^L219M^ increases lactate tolerance in *Methylomonas* sp. JHM80 and DH-1. Growth curves of the indicated strains cultured in NMS media containing lactate (**A**, **B**, **D**) and in control NMS media (**C**, **E**). Twenty percent (v/v) methane was used as the carbon source in all conditions. Error bars represent standard deviation (SD) from three biological replicates. (**F**) Schematic illustration of AlaRS protein domains highlighting the position of Leu219.

To produce lactic acid from methane, we previously developed a lactate-tolerant strain, JHM80, through ALE of a methanotroph, *Methylomonas* sp. DH-1 [19]. While JHM80 was able to tolerate up to 8 g/l lactate, we enhanced lactate tolerance further by subjecting JHM80 to another round of ALE. JHM80 cells were grown in media containing gradually increasing lactate concentrations over 32 days, resulting in the development of JHM102 strain. This new strain could tolerate up to 10 g/l lactate in a medium neutralized to pH 6.8, whereas JHM80 could not survive under the same conditions (Fig. 1A). Through whole-genome sequencing of the JHM80 and JHM102 strains, we discovered a single-point mutation C655A in the *alaRS* gene of JHM102. This mutation led to the L219M substitution in the catalytic domain of AlaRS that consists of an N-terminal catalytic domain for aminoacylation, a tRNA recognition domain, an editing domain, and a C-Ala domain (Fig. 1F) [9, 34]. To validate the effect of L219M mutation on lactate tolerance, we introduced the mutation into JHM80 and wild-type DH-1 strains. The introduction of *alaRS*^L219M^ mutation led to an increase in lactate tolerance in both strain backgrounds, even though they had different levels of lactate tolerance initially (Fig. 1B and D). The JHM80 AlaRS^L219M^ mutant strain demonstrated the ability to survive in the presence of 10 g/l lactate, like JHM102 (Fig. 1B). Likewise, the DH-1 AlaRS^L219M^ mutant could survive in the presence of 1 g/l lactate, whereas the DH-1 wild-type strain, which experienced growth inhibition at concentrations as low as 0.15 g/l lactate [19], could not survive under the same conditions (Fig. 1D). In the control NMS media, all tested strains showed similar growth, indicating that the *alaRS*^L219M^ mutation specifically affects the cell growth in the presence of lactate (Fig. 1C and E).

### L219M mutation in AlaRS enhances both the activity and fidelity of the enzyme

**Figure 2.**
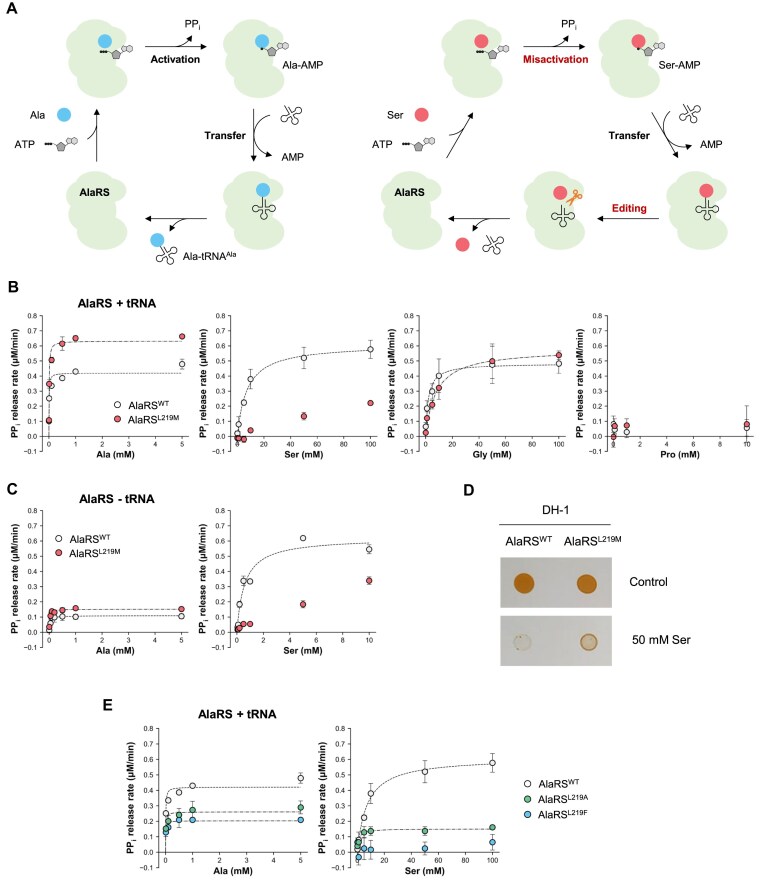
AlaRS^L219M^ mutation enhances both the activity and fidelity of the enzyme. (**A**) Schematic representation of enzymatic reactions catalyzed by AlaRS using either the cognate substrate Ala or the non-cognate substrate Ser. (**B**) PPi release assay evaluating the enzyme activity of wild-type AlaRS (AlaRS^WT^) and the AlaRS^L219M^ mutant in the presence of tRNA^Ala^ and various amino acids. (**C**) PPi release assay of AlaRS and AlaRS^L219M^ in the absence of tRNA using Ala or Ser as substrates. (**D**) Spotting assay comparing the growth of *Methylomonas* sp. DH-1 strains expressing AlaRS^WT^ or AlaRS^L219M^ on NMS minimal medium and NMS supplemented with 50 mM Ser. (**E**) PPi release assay of AlaRS^L219F^ and AlaRS^L219A^ in the presence of tRNA^Ala^ with Ala or Ser. The AlaRS^WT^ data shown for comparison correspond to panel (B). Error bars represent SD (*n* = 3).

Given that the L219M mutation is located within the catalytic domain of AlaRS, we hypothesized that this mutation may impact the aminoacylation activity of the enzyme. To explore this possibility, we conducted an *in vitro* aminoacylation assay in the presence of tRNA^Ala^ to compare the enzymatic activities of wild-type AlaRS and AlaRS^L219M^. This assay measures PPi released during the amino acid activation step, serving as an indicator of aminoacylation activity (Fig. 2A).

We evaluated a range of substrates, including Ala (the cognate substrate), Ser and Gly (non-cognate substrates), and Pro (used as a negative control). When Ala was used as a substrate, AlaRS^L219M^ exhibited an increase in *k*_cat_/*K*_m_ compared with the wild-type AlaRS, indicating enhanced catalytic efficiency toward Ala (Fig. 2B and Table 1). Wild-type AlaRS also demonstrated activity toward the non-cognate substrates Ser and Gly, although with significantly reduced catalytic efficiencies—only 0.2%–0.5% of the efficiency observed with Ala (Fig. 2B and Table 1). Interestingly, however, the AlaRS^L219M^ mutant demonstrated no activity toward Ser except at extremely high substrate concentrations, while its activity toward Gly remained comparable to that of the wild type (Fig. 2B and Table 1). No enzymatic activity was observed for either AlaRS or AlaRS^L219M^ when Pro was used as a substrate (Fig. 2B).

**Table 1.** Kinetic characterization of wild-type and mutant AlaRS in PPi release assays

Substrate	Alanine				Serine				Glycine			
	*K*_m_ (mM)	*k*_cat_ (min^−1^)	*k*_cat_/*K*_m_(mM^−1^ min^−1^)	*k*_cat_/*K*_m_ ratio^a^	*K*_m_ (mM)	*k*_cat_ (min^−1^)	*k*_cat_/*K*_m_ (mM^−1^ min^−1^)	*k*_cat_/*K*_m_ ratio	*K*_m_ (mM)	*k*_cat_ (min^−1^)	*k*_cat_/*K*_m_ (mM^−1^ min^−1^)	*k*_cat_/*K*_m_ ratio
AlaRS^WT^	0.007 ± 0.001	0.416 ± 0.013	57.744 ± 8.036	1	7.308 ± 1.655	0.612 ± 0.080	0.085 ± 0.015	0.002	2.055 ± 0.920	0.477 ± 0.101	0.273 ± 0.143	0.005
AlaRS^L219M^	0.009 ± 0.001	0.621 ± 0.022	66.238 ± 6.943	1	ND^b^	ND	–	–	7.906 ± 3.276	0.573 ± 0.088	0.078 ± 0.022	0.001
AlaRS^V204A L219M^	0.145 ± 0.039	0.420 ± 0.012	3.024 ± 0.701	1	1.131 ± 0.337	0.373 ± 0.017	0.353 ± 0.122	0.117	–^c^	–	–	–
AlaRS^V204L L219M^	ND	ND	–	–	ND	ND	–	–	ND	ND	–	–
AlaRS^L219F^	0.007 ± 0.003	0.201 ± 0.020	32.486 ± 13.806	1	ND	ND	–	–	–	–	–	–
AlaRS^L219A^	0.009 ± 0.006	0.259 ± 0.050	35.274 ± 15.896	1	1.027 ± 0.278	0.149 ± 0.023	0.153 ± 0.048	0.004	–	–	–	–
AlaRS^V204A^	0.003 ± 0.001	0.190 ± 0.040	56.687 ± 6.409	1	15.914 ± 1.372	1.898 ± 0.121	0.119 ± 0.006	0.002	–	–	–	–
AlaRS^V204L^	0.005 ± 0.001	0.181 ± 0.027	39.220 ± 12.110	1	17.030 ± 6.470	0.370 ± 0.054	0.024 ± 0.008	0.001	–	–	–	–
AlaRS_429_	0.006 ± 0.001	0.105 ± 0.003	19.444 ± 4.518	1	0.164 ± 0.027	0.257 ± 0.019	1.584 ± 0.146	0.081	–	–	–	–
AlaRS_429_^L219M^	0.003 ± 0.001	0.141 ± 0.009	43.030 ± 11.637	1	ND	ND	–	–	–	–	–	–
AlaRS^WT^ (without tRNA)	0.028 ± 0.015	0.107 ± 0.022	4.301 ± 1.180	1	0.579 ± 0.060	0.615 ± 0.016	1.071 ± 0.121	0.235	–	–	–	–
AlaRS^L219M^ (without tRNA)	0.016 ± 0.007	0.150 ± 0.009	10.835 ± 5.053	1	ND	ND	–	–	–	–	–	–

Data are presented as mean ± SD (*n* = 3).

^a^The *k*_cat_/*K*_m_ ratios for reactions involving non-cognate Ser and Gly were calculated normalized to those with Ala for each enzyme variant.

^b^ND, *k*_cat_ and *K*_m_ were not determined due to the low activity

^c^–, not calculated or not conducted.

Although the L219M mutation resides within the catalytic domain, we sought to determine whether the reduced reactivity of AlaRS^L219M^ toward Ser was affected by the enzyme’s editing activity. To investigate this, we conducted assays using truncated variants, AlaRS_429_ and AlaRS_429_^L219M^, which consist of residues 1–429 and lack the editing domain (Fig. 1F). AlaRS_429_ retained activity toward both Ala and Ser, similar to the full-length wild-type AlaRS (Supplementary Fig. S1 and Table 1). In contrast, AlaRS_429_^L219M^ displayed a similar activity profile to its full-length counterpart, showing enhanced activity toward Ala and no detectable activity toward Ser when compared to AlaRS_429_ (Supplementary Fig. S1 and Table 1). These results suggest that AlaRS^L219M^ can discriminate against Ser during the aminoacylation step, independently of the editing domain.

The aminoacylation assay we used measures PPi release during amino acid activation. However, the overall reaction rate can also be influenced by the subsequent aminoacyl transfer to tRNA, since release of aminoacyl-tRNA from the enzyme is necessary for new catalytic cycles to begin (Fig. 2A). To specifically assess the contribution of the amino acid activation step, we conducted the assay in the absence of tRNA, thereby eliminating any effects from the aminoacyl transfer process. In the absence of tRNA, the rate of PPi release decreased for both wild-type AlaRS and the AlaRS^L219M^ mutant when reacting with Ala, but increased when reacting with Ser, relative to reactions performed in the presence of tRNA (Fig. 2C and Table 1). This shift may reflect more efficient release of the aminoacyl adenylate intermediate from the non-cognate substrate (Ser) in the absence of the aminoacyl transfer step. Despite these changes in reaction rates, the overall trend remained consistent: AlaRS^L219M^ showed higher activity toward Ala and significantly reduced activity toward Ser compared to the wild-type enzyme (Fig. 2C and Table 1). These results clearly demonstrate that the enhanced substrate discrimination conferred by the L219M mutation occurs at the amino acid activation step, rather than during aminoacyl transfer.

To validate the enhanced fidelity of AlaRS^L219M^ in discriminating against Ser, we investigated the effect of elevated Ser concentrations, which could be toxic to the cells partly by increasing the likelihood of Ser misincorporation during translation. DH-1 strains expressing either AlaRS^WT^ and AlaRS^L219M^ showed similar growth rate on standard NMS media (Fig. 2D). However, upon exposure to 50 mM Ser, both strains experienced growth inhibition. Notably, the DH-1 AlaRS^L219M^ mutant strain demonstrated higher tolerance compared to the wild-type strain (Fig. 2D). This finding supports the conclusion that AlaRS^L219M^ reduces Ser mischarging to tRNA^Ala^, thereby enhancing translational accuracy.

Given that the L219M mutation alters substrate specificity, we conducted further experiments to assess the effects of substituting Leu219 with either a smaller residue (Ala) or a larger one (Phe). Aminoacylation assays revealed that AlaRS^L219F^, like AlaRS^L219M^, did not react with Ser, suggesting that the L219F mutation similarly restricts Ser activation (Fig. 2E and Table 1). In contrast, AlaRS^L219A^ retained reactivity toward Ser, resembling the wild-type enzyme (Fig. 2E and Table 1). These findings indicate that point mutations at position 219 of AlaRS can modulate both catalytic activity and substrate specificity.

### Overall structure of AlaRS^L219M^ in complex with both ATP and Ala

**Figure 3.**
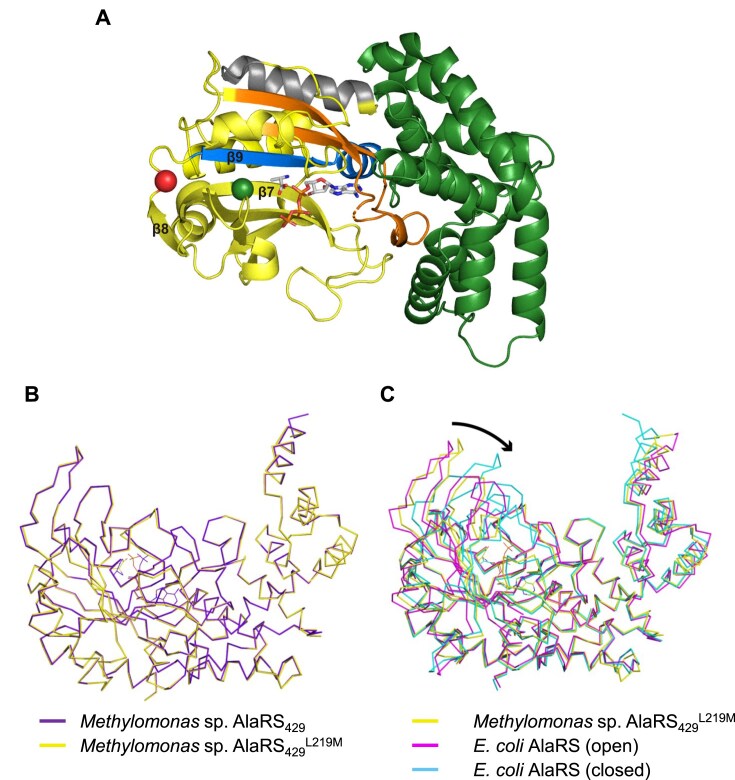
Overall structure of *Methylomonas* sp. AlaRS_429_^L219M^ and structural comparison among *Methylomonas* sp. AlaRS_429_, its L219M variant (AlaRS_429_^L219M^), and *E. coli* AlaRS. (**A**) Ribbon diagram of AlaRS_429_^L219M^ showing bound ATP and Ala as stick models. The catalytic domain is colored yellow and the tRNA recognition domain green. The Cα atoms of Met219 and Val204 are highlighted with red and green spheres, respectively. Motifs 1, 2, and 3 are colored in gray, orange, and blue, respectively. (**B**) Structural superposition of AlaRS_429_ (purple) and AlaRS_429_^L219M^ (yellow). (**C**) Superposition of AlaRS_429_^L219M^ with *E. coli* AlaRS in both the closed (cyan, PDB: 3HXU) and open (magenta, PDB code: 3HY1) conformations. The arrow indicates the ∼11 Å conformational shift between the open and closed states of *E. coli* AlaRS.

To get structural explanation why the L219M mutation prevents AlaRS from misactivating Ser to produce seryl-adenylate, we determined the crystal structures of both the wild-type and L219M mutant AlaRS proteins in complex with ATP and Ala (Supplementary Table S5). The N-terminal fragment of AlaRS corresponding to residues 1–429 was used for successful crystallization. Their structures are referred to as AlaRS_429_ for the wild type and AlaRS_429_^L219M^ for the L219M mutant. AlaRS_429_ and AlaRS_429_^L219M^ consist of the N-terminal nine-stranded β-sheet structure harboring the catalytic site (residues 1–241) and the C-terminal α-helix bundle (residues 242–429) responsible for tRNA recognition (Fig. 3A). These domains are often collectively referred to as the aminoacylation domain and contain the three signature motifs of class II AARSs, located at residues 4–20, 60–96, and 223–238, respectively [8]. Both structures are virtually identical to each other, with a root-mean-square deviation value of 0.242 Å for all Cα atoms, indicating that the L219M mutation has little structural effect (Fig. 3B). Hereafter, the structure of AlaRS_429_^L219M^ was employed to describe the binding modes of ATP and Ala since electron density for Ala was clearer in this structure.

The presence of ATP and Ala in their intact forms indicates that this structure depicts the conformational state before activation. The closest structural homolog of AlaRS_429_^L219M^, identified using the DALI server, is *E. coli* AlaRS [35]. Multiple crystal structures of *E. coli* AlaRS, both in its apo form and in complex with intermediate analogs, have revealed that the enzyme undergoes a distinct conformational change from an “open” to a “closed” state. When the structure of AlaRS_429_^L219M^ is superposed onto those of *E. coli* AlaRS (Fig. 3C), it was obvious that AlaRS_429_^L219M^ resembles the open structure of the apo state rather than the closed structure depicting the conformational state after aminoacylation. Therefore, it appears that the closed conformation is achieved not on ligand binding but during/after the aminoacylation reaction.

### The binding modes of ATP and Ala in AlaRS_429_^L219M^ and local structures around residue 219 in AlaRS_429_ and AlaRS_429_^L219M^

**Figure 4.**
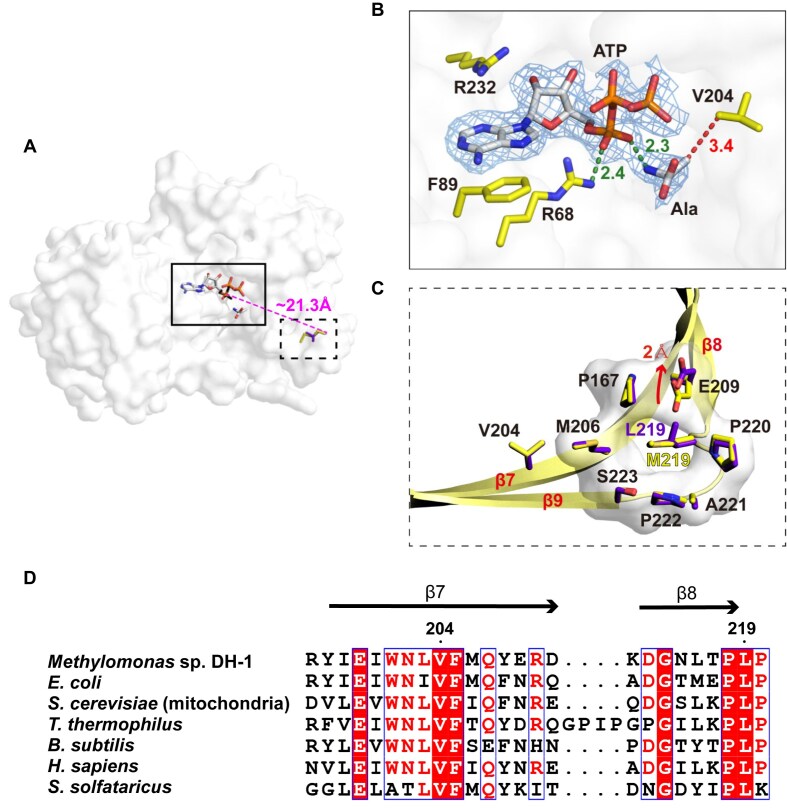
The binding modes of ATP and Ala and local structure around Leu219/Met219. (**A**) Surface representation of AlaRS_429_^L219M^ showing ATP, Ala, and Leu219/Met219 as sticks. A magenta dotted line indicates the distance between the phosphorus atom of the α-phosphate and the Cα atoms of Leu219/Met219. (**B**) Close-up view of the boxed region showing interactions between active site residues (yellow) and ATP/Ala ligands (white). Final *2Fo–Fc* electron density maps (blue) are contoured at 1*σ* for ATP and Ala. Distances (Å) are labeled; green and red dotted lines represent polar and hydrophobic interactions, respectively. (**C**) Close-up view of the dotted boxed region showing the local structure around Leu219/Met219. Residues from AlaRS_429_ and AlaRS_429_^L219M^ are shown as purple and yellow sticks, respectively. (**D**) Multiple sequence alignment of the β7 and β8 strands of AlaRS. Residue numbers correspond to *Methylomonas* sp. DH-1 AlaRS. GenBank accession numbers: WP_197495997.1 (*Methylomonas* sp. DH-1), WP_105477077.1WP_197495997.1 (*E. coli*), GHM91772.1 (*Saccharomyces cerevisiae*), WP_223968742.1 (*Thermus thermophilus*), WP_262119243.1 (*Bacillus subtilis*), KAI2579449.1 (*Homo sapiens*), and WP_009990629.1 (*Sulfolobus solfataricus*).

The active site cavity is situated on one face of the N-terminal nine-stranded β-sheet, the other face of which is covered by α-helices (Fig. 4A). There are two representative interactions between ATP and the active site (Fig. 4B). First, the adenine base is stacked between Phe89 in motif 2 and Arg232 in motif 3, which anchors ATP in the active site. Second, the α-phosphate group forms electrostatic interactions with the guanidinium side chain of Arg68 in motif 2, which contributes to stabilizing negative charge accumulating at the α-phosphate during aminoacylation reaction [36, 37]. Ala is positioned in the vicinity of the α-phosphate of ATP. The α-NH_3_^+^ group of Ala interacts with the α-phosphate group of ATP and its methyl side chain forms hydrophobic contact with Val204 (Fig. 4A and B).

Leu219 in AlaRS_429_ and Met219 in AlaRS_429_^L219M^ are not residues constituting the active site. Their Cα atoms are ∼21.3 Å away from the phosphorus atom of the α-phosphate group of ATP (Fig. 4A). According to the superposed structures of AlaRS_429_ and AlaRS_429_^L219M^, the side chains of Leu219 and Met219 are situated in a hole formed by Pro167, Met206, Glu209, Pro220, Ala221, Pro222, and Ser223 (in motif 3) (Fig. 4C). The only structural difference in the hole between AlaRS_429_ and AlaRS_429_^L219M^ is the rotamer change of Glu209, which is since methionine has a linear side chain, but leucine has a branched side chain.

### Confirmation of the role of Val204 to discriminate Ala and Ser in AlaRS^L219M^

The distance between the methyl side chain of Ala and Val204 is 3.4 Å, which suggests that if Ser were positioned in the same space, the extra hydroxyl group of Ser would clash with Val204 (Fig. 4B). This perspective is consistent with experimental findings indicating that AlaRS^L219M^ did not use Ser as a substrate (Fig. 2A and Table 1). However, there is no difference in the position of Val204 observed in the crystal structures of both AlaRS_429_ and AlaRS_429_^L219M^ (Fig. 4C). This discrepancy prompts the question of why the wild-type AlaRS can effectively use Ser as a substrate, or in simpler terms, how Val204 in the wild type does not cause any steric hindrance with the hydroxyl group of Ser. Leu219 in AlaRS_429_ and Met219 in AlaRS_429_^L219M^ contact with the C-terminal segment (residues 206–209) of the β7 strand harboring Val204 (Fig. 4C), and thus the amino acid identity at position 219 could affect the dynamic motion of Val204. The Val204 and Leu219 residues are conserved in AlaRS orthologs from bacteria to humans (Fig. 4D).

**Figure 5.**
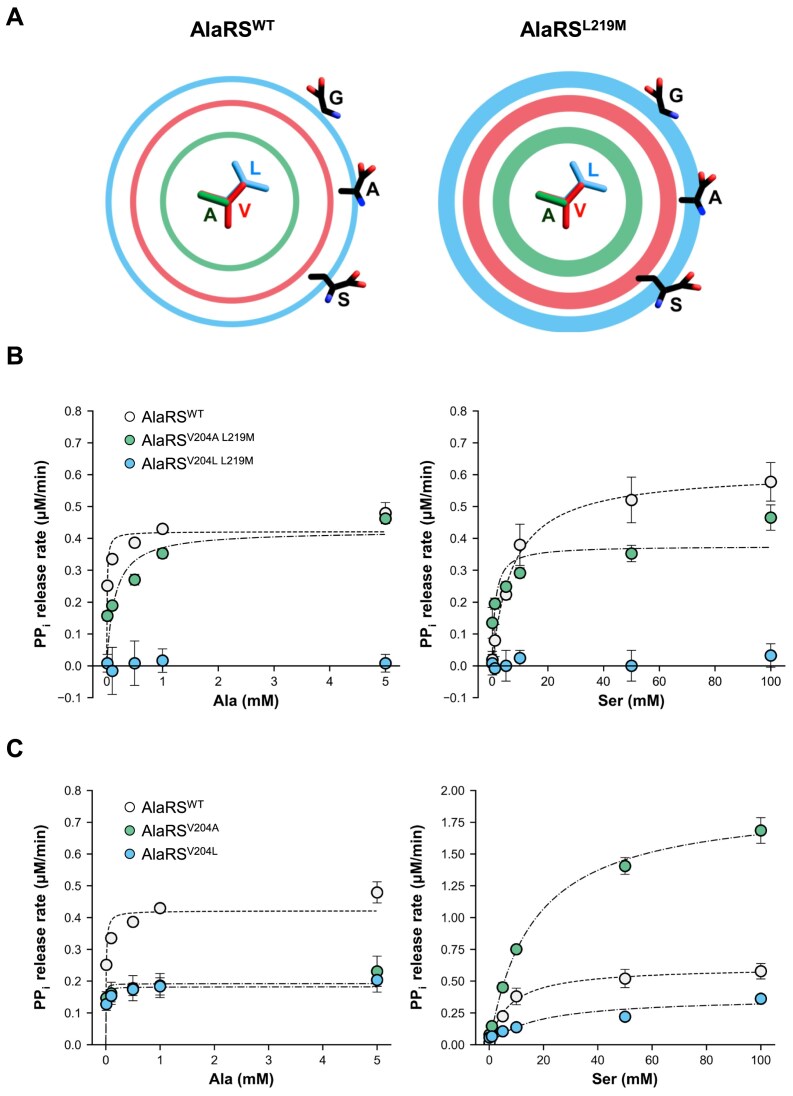
Influence of Val204 flexibility on substrate binding. (**A**) Schematic illustration of the van der Waals excluded volumes for Ala204, Val204, and Leu204. Ring diameters represent the van der Waals sizes of Ala (green), Val (red), and Leu (blue), while ring thickness reflects side chain flexibility, inferred from the *B*-factors of AlaRS^WT^ and AlaRS^L219M^. (**B**) PPi release assay for AlaRS^V204A L219M^ and AlaRS^V204L L219M^ double mutants in the presence of tRNA^Ala^, using Ala and Ser as substrates. (**C**) PPi release assay of AlaRS^V204A^ and AlaRS^V204L^ single mutants under identical conditions. AlaRS^WT^ data are shown for comparison and correspond to results in Fig. 2B. Error bars represent SD (n = 3).

Crystallographically determined *B*-factors, which account for atom displacements due to thermal motion and conformational disorder, are widely recognized as valuable indicators for estimating protein structure flexibility [38–40]. Various factors, such as crystal packing interactions, data collection temperatures, and refinement methodologies, can influence the *B*-factors of proteins [39]. Crystals of AlaRS_429_ and AlaRS_429_^L219M^ were isomorphous, their diffraction data were collected at 100 K, and their models were refined in the same way. Therefore, the *B*-factor comparison between AlaRS_429_ and AlaRS_429_^L219M^ provides information on the flexibility change of Val204 induced by the L219M point mutation. Interestingly, the *B*-values of Cα atoms of Val204 in AlaRS_429_ and AlaRS_429_^L219M^ are 19.96 and 26.68 Å^2^, respectively, suggesting that Val204 is more flexible in AlaRS_429_^L219M^ than in AlaRS_429_. To confirm the *B*-factor difference, normalized *B*′-factors of Val204 in both proteins were calculated using the following equation: *B*′= (*B −*〈*B*〉/*σ*(*B*), where 〈*B*〉 and *σ*(*B*) represent the average *B*-factor value of all Cα atoms and the SD of the *B*-factors, respectively [39]. Consistently, the Cα atom of Val204 in AlaRS_429_^L219M^ has a higher *B*′-factor (−0.85 Å^2^) than that in AlaRS_429_ (−1.14 Å^2^). According to the Lennard-Jones model, even small changes in distance can lead to pronounced impacts on van der Waals repulsive forces [41, 42]. Given the higher *B*-factor of Val204 in AlaRS_429_^L219M^, there would be van der Waals repulsion between Val204 and Ser in the active site. That is, the more dynamic Val204 of AlaRS_429_^L219M^ invades the space intended for the extra hydroxyl group of Ser, whereas the relatively static Val204 in AlaRS_429_ allows the binding of Ser without inducing clash with the hydroxyl group (Fig. 5A).

Based on the results of structure determination and analysis of *B*-factor value, the L219M mutation appears to subtly increase the flexibility of the otherwise static Val204, potentially hindering Ser access. To examine whether the Val204-mediated steric clash is critical in substrate discrimination, we introduced additional mutations at position 204 in the AlaRS^L219M^ background—substituting Val with either a smaller residue (Ala) or a larger one (Leu). We hypothesized that introducing a smaller residue at position 204 would create enough space to accommodate the extra hydroxyl group of Ser, thereby reducing substrate discrimination (Fig. 5A). In contrast, substituting with a larger residue was expected to introduce steric hindrance, potentially blocking the binding of even smaller substrates like Ala and Gly. The resulting double mutants AlaRS^V204A L219M^ and AlaRS^V204L L219M^ were examined for their aminoacylation activities toward Ala and Ser *in vitro*. Remarkably, the additional V204A mutation acted as a suppressor of the L219M effect, reducing activity toward Ala and restoring activity toward Ser (Fig. 5B and Table 1). Consistent with the role of Val204 in blocking the access of Ser, AlaRS^V204A L219M^ exhibited a higher *k*_cat_/*K*_m_ value for Ser compared to the wild-type AlaRS (Fig. 5B and Table 1). In contrast, the V204L mutation completely abolished the activity of AlaRS^L219M^ toward Ser, Ala, and even Gly (Fig. 5B, Table 1, and Supplementary Fig. S2). These results suggest that the small side chain of Ala at position 204, despite the increased flexibility introduced by the L219M mutation, is insufficient to obstruct the extra hydroxyl group of Ser. Conversely, the larger Leu side chain at position 204, when combined with the enhanced flexibility from the L219M mutation, creates steric hindrance that blocks access for all tested substrates (Fig. 5A).

To evaluate the individual effect of the Val204 mutation, we also tested the enzymatic activities of AlaRS^V204A^ and AlaRS^V204L^ toward Ala and Ser (Fig. 5C and Table 1). Both variants showed reduced *k*_cat_/*K*_m_ values for Ala compared to wild-type AlaRS, indicating that Val204 contributes to the catalytic efficiency of the enzyme (Table 1). Regarding Ser activity, AlaRS^V204A^ exhibited higher *k*_cat_/*K*_m_ values, while AlaRS^V204L^ showed lower values relative to the wild-type AlaRS (Fig. 5C and Table 1). These findings are consistent with previous results from the AlaRS^V204A L219M^ and AlaRS^V204L L219M^, reinforcing the idea that at position 204, an Ala substitution facilitates Ser binding, whereas a Leu substitution impedes it.

### AlaRS^L219M^ mutation eliminates lactyltransferase activity

To better understand how the AlaRS^L219M^ mutation increase lactate tolerance, we compared proteomes of *Methylomonas* sp. DH-1 strains expressing either AlaRS^WT^ or AlaRS^L219M^ under both normal and lactate stress conditions (0.15 g/l lactate). Notable differences in proteomic profiles were observed under both conditions, indicating that alterations in AlaRS activity and fidelity can affect overall protein expression patterns (Supplementary Fig. S3A and B). Although several proteins showed differential expression between the DH-1 AlaRS^WT^ and DH-1 AlaRS^L219M^ strains, no specific proteins could be directly linked to lactate tolerance. This suggests that the observed tolerance may result from qualitative shifts in proteome composition rather than changes in the abundance of individual proteins.

**Figure 6.**
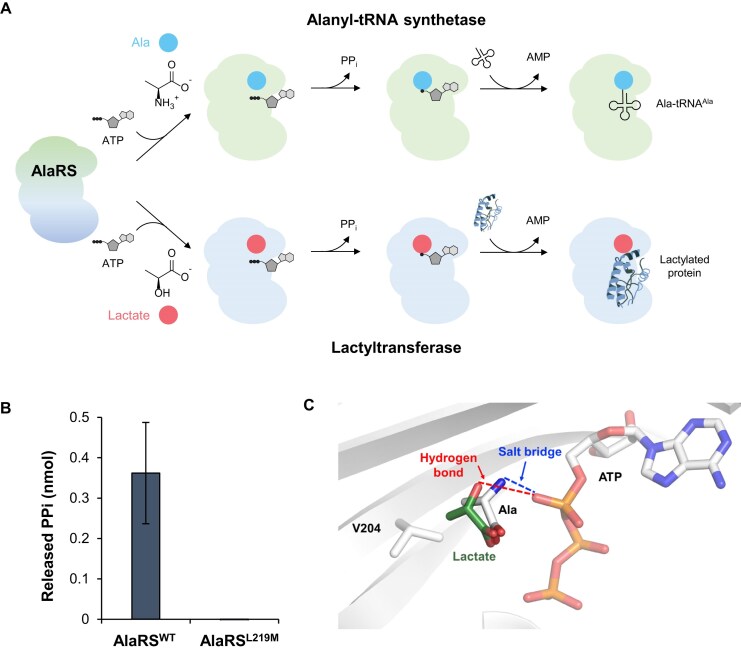
AlaRS^L219M^ mutation eliminates lactyltransferase activity of AlaRS. (**A**) Schematic representation comparing the canonical alanyl-tRNA synthetase activity of AlaRS with its proposed lactyltransferase activity (**B**) Quantification of lactate activation by AlaRS^WT^ and the AlaRS^L219M^. Enzymatic reactions were performed with 10 mM lactate for 15 min, and the released PPi was measured as an indicator of activity. Error bars represent SD (n = 3). (**C**) Predicted structural model of lactate binding within the AlaRS active site compared to Ala. Salt bridge and hydrogen bonding interactions are shown as dotted lines.

AlaRS has recently been identified as not only a tRNA synthetase but also a lactate sensor and lactyltransferase, mediating global lysine lactylation [24]. Analogous to amino acid activation during tRNA aminoacylation, lactate, which structurally resembles Ala, can be adenylated by AlaRS. This activated intermediate can then be transferred to Lys residues on target proteins (Fig. 6A). Based on this, we hypothesized that the lactate tolerance conferred by the AlaRS^L219M^ mutation might be linked to its altered lactylation activity. To investigate this, we performed a lactylation assay that measures PPi release during lactate activation via ATP-dependent adenylation. In the presence of 10 mM lactate, PPi release was detected in reactions with AlaRS^WT^ but not with AlaRS^L219M^ (Fig. 6B). These findings suggest that AlaRS^L219M^ has a diminished ability to activate lactate, similar its reduced reactivity toward Ser.

From a structural standpoint, the loss of lactyltransferase activity in AlaRS^L219M^ can likely be attributed to the increased flexibility of Val204. Although Ala and lactate share similar structures, their key difference lies in the presence of an α-amino group (α-NH_3_^+^) in Ala, whereas lactate contains a hydroxyl group (–OH) at the corresponding position (Fig. 6A). In the crystal structure of AlaRS_429_^L219M^ (Fig. 4B), the α-amino group of Ala forms a salt bridge with the α-phosphate of ATP. However, the hydroxyl group of lactate forms a hydrogen bond, which is generally weaker than an ionic interaction [43]. This weaker interaction likely positions lactate slightly farther from the α-phosphate and closer to Val204 (Fig. 6C). Given this shift, the increased flexibility of Val204 in AlaRS^L219M^ could result in a steric clash with the methyl side chain of lactate, thereby impeding lactate binding. Given the critical role of post-translational modifications in regulating protein function and the fact that lactylation can impair protein activity, it is likely that the enhanced lactate tolerance conferred by AlaRS^L219M^ arises from preventing excessive protein lactylation under lactate stress. This finding indicates that the enhanced substrate fidelity conferred by the AlaRS^L219M^ mutation extends beyond amino acids to include lactate, thereby contributing to increased cellular tolerance to high concentrations of both serine and lactate.

## Discussion

It has been proposed that the Ser misactivation is an unavoidable occurrence within the nature-designed catalytic mechanisms of AlaRS across all domains of life [12, 14, 34]. A previous structural study of *E. coli* AlaRS complexed with either the reaction intermediate alanyl-adenylate or intermediate analog (Ala-SA, Ser-SA, and Gly-SA) revealed that all three ligands bind within the active site through favorable interactions between the α-amino group of the amino acid moiety and the carboxylate side chain of Asp235 (corresponding to Asp225 in DH-1 AlaRS) [8]. Notably, in the case of Ser-SA, both the side chain hydroxyl group and the α-amino group of the seryl moiety interact with Asp235, contributing to the strong binding of Ser-SA within the active site. As a result, once an amino acid—whether Ala or Ser—enters the active site and forms these stabilizing interactions, the activation reaction proceeds to generate the corresponding aminoacyl-adenylate. At this stage, the enzyme is no longer able to discriminate between the correct and incorrect substrates. If this is the case, the generation of Ser-tRNA^Ala^, which leads to the misincorporation of Ser in place of Ala during protein synthesis, is inevitable once Ser binds to the active site of AlaRS. Therefore, to prevent mistranslation caused by the Ser misactivation, it is essential to eliminate the mischarged Ser-tRNA^Ala^ molecules either by the editing domain within AlaRS or by a separate editing protein such as AlaXP [10].

In this study, we provided evidence that the AlaRS^L219M^ mutation, originally identified as the key mutation conferring lactate tolerance in the methanotrophic bacterium JHM102, enhances the fidelity of AlaRS by effectively preventing Ser misactivation. While the enzyme activity assay used in this study, which measures PPi release during the amino acid activation step, does not yield precise kinetic parameters for the activation reaction alone, it reflects the overall catalytic process of AlaRS, including activation, transfer, and editing, particularly when non-cognate substrates are involved. Remarkably, AlaRS^L219M^ exhibited no detectable activity toward Ser in the absence of the editing domain or tRNA, indicating that this variant is capable of discriminating against Ser at the activation step. Structural analysis of both wild-type and L219M mutant AlaRS_429_ further supports this hypothesis, revealing that the L219M substitution may inhibit Ser binding by increasing the flexibility of the neighboring residue, Val204. This discovery marks a significant milestone, as the L219M substitution represents the first reported mutation that enhances the fidelity of AlaRS, contrasting with the many previously reported mutations leading to editing deficiencies [11, 14]. Furthermore, it is particularly noteworthy that this enhanced fidelity is conferred by a single-point mutation within the aminoacylation domain, rather than the editing domain. In a prior investigation, Asp235 of *E. coli* AlaRS, an essential residue involved in binding seryl-adenylate within the alanyl-adenylate binding pocket, was substituted with various amino acids. However, none of the substitutions enabled discrimination between alanyl-adenylate and seryl-adenylate, highlighting the unique fidelity-enhancing effect of the L219M mutation [8].

Unlike the previous study determining the structures of the post-activation states [8], our structural studies about both AlaRS_429_ and AlaRS_429_^L219M^ in complex with Ala and ATP depicted, for the first time, a visualization of the spatial arrangement of both substrates prior to the activation reaction. Structural analyses of the pre-reaction state revealed that the L219M mutation increases the flexibility of Val204. This enhanced flexibility enables Val204 to function as a “molecular discriminator,” selectively blocking the binding of Ser to the active site of AlaRS_429_^L219M^ while still permitting the accommodation of Ala. Confronting this simple mechanism preventing the misactivation of Ser, we encounter an evolutionary puzzle: why has this specific point mutation not been naturally selected over billions of years of AlaRS evolution? In other words, why have Leu219 and Val204 been conserved in AlaRS orthologs? (Fig. 4D). A highly plausible explanation has already been proposed: the editing domain or proteins likely emerged concurrently with the early development of AlaRS [8]. If this is the case, the evolutionary pressure to introduce the L219M mutation into AlaRS would be negligible. As the L219M mutation does not prevent glycine misactivation, the editing domain may still be necessary for precise translation. Furthermore, since the L219M mutation abolishes the lactyltransferase activity of AlaRS, which is essential for lysine lactylation as post-translational modification that connects cellular metabolism to protein function, retaining the editing machinery may have been more advantageous than acquiring the L219M mutation [24–26]. In addition, AARSs have diverse roles beyond amino acid–tRNA linkage. For instance, cysteinyl-tRNA synthetases (CysRSs) function as essential cysteine persulfide synthetases [44]. Likewise, mitochondrial tyrosyl-tRNA synthetase (TyrRS) in *Neurospora crassa* serves as a splicing factor for mitochondrial group I introns, and human TyrRS undergoes proteolytic cleavage to generate fragments with cytokine activity [45–47]. Furthermore, bacterial transfer–messenger RNA, fused with tRNA^Ala^ and mRNA encoding tag peptide, is charged by AlaRS to participate in *trans*-translation, facilitating the degradation of nascent polypeptides from damaged mRNA [48, 49]. Many AARSs also form multienzyme complexes through interactions with other AARSs and proteins [1]. This variety of functions may provide the evolutionary advantage in maintaining other domains despite potential fidelity increases from simple mutations affecting the catalytic domain.

It is intriguing that the AlaRS^L219M^ mutation, which might have been impossible to design rationally, emerged spontaneously during ALE aimed at enhancing lactate tolerance. Remarkably, the isolation of this mutant was possible because of the dual functionality of AlaRS, serving both as an alanyl-tRNA synthetase and as a lactyltransferase. The high-fidelity AlaRS^L219M^ mutation reduces the activation of both Ser and lactate in its dual enzymatic roles. DH-1 strains expressing either wild-type AlaRS or the AlaRS^L219M^ mutant displayed distinct proteomic profiles under both normal and lactate stress conditions. However, identifying specific proteins directly linked to lactate tolerance among the differentially expressed proteins proved challenging. Under lactate stress, widespread protein lactylation may alter protein function, potentially leading to growth inhibition. Therefore, the lactate tolerance conferred by the AlaRS^L219M^ mutant may stem from decreased lactylation of target proteins, rather than from changes in overall proteome composition due to enhanced translational fidelity. Further in-depth studies are needed to identify the proteins lactylated by AlaRS and to determine which of them are critical in lactylation-dependent lactate toxicity.

The role of AlaRS as a lactyltransferase has only recently been clarified, and the impact of lactylation has been identified in a limited number of target proteins. The lactylation process has been associated with lactate-induced tumorigenesis. For example, AlaRS-dependent lactylation of p53 inhibits its DNA-binding activity, diminishing its tumor-suppressive functions [24]. Additionally, lactylation by AlaRS activates the YAP–TEAD1 complex within the Hippo signaling pathway, promoting proliferation in gastric cancer cells [25]. In another instance, intracellular hypoxia triggers the lactylation of mitochondrial proteins such as PDHA1 in the pyruvate dehydrogenase complex and carnitine palmitoyltransferase 2, leading to decreased oxidative phosphorylation [26]. The high-fidelity AlaRS mutant identified in our study could help elucidate the role of lactylation in various biological processes.

In conclusion, the spontaneous emergence of the AlaRS^L219M^ mutant not only reveals an elegant mechanism of fidelity enhancement within the aminoacylation domain but also provides a powerful tool to understand the multifaceted roles of AlaRS—particularly its emerging function as a lactyltransferase—in cellular physiology and disease.

## Supplementary Material

gkaf462_Supplemental_File

## Data Availability

The atomic coordinates and structure factors of the final models have been deposited in the Protein Data Bank (www.pdb.org) with ID codes 9JC7 (the wild type) and 9JDN (the L219M mutant). The mass spectrometry proteomics data have been deposited to the ProteomeXchange Consortium via the PRIDE partner repository with the dataset identifier PXD044904 [50].
