# Supplementary material for: Unlocking the serine mischarging paradox and inhibiting lactyltransferase activity of AlaRS by a single-point mutation
Source: Nucleic Acids Res. 2025 Jun 6;53(11):gkaf462. doi: 10.1093/nar/gkaf462 (PMC12143591; doi:10.1093/nar/gkaf462)
Supplement: gkaf462_Supplemental_File [file gkaf462_supplemental_file.pdf]

Supplementary data

Unlocking the serine mischarging paradox and inhibiting lactyltransferase activity of AlaRS by a single-point mutation

Wooyoung Park[1,#], Se-Young Son[2,#], Joonyeop Yi[3,4], SeungWoo Cha[1], Hankyeol Moon[1], Minyoung Kim[3,4], Sangho Ji[5], Wookyung Yu[5], , Changmin Sung[3], Sun-Shin Cha[2,6]*, and Ji-Sook Hahn[1,4]*

[1] Department of Chemical and Biological Engineering, Institute of Chemical Processes, Seoul National University, Seoul, 08826, Republic of Korea

[2] Department of Chemistry & Nanoscience, Ewha Womans University, Seoul, 03760, Republic of Korea

[3] Doping Control Center, Korea Institute of Science and Technology, Seoul, 02792, Republic of Korea

[4] Interdisciplinary Program for Biochemical Engineering and Biotechnology, Seoul National University, Seoul, 08826, Republic of Korea

[5] Department of Brain Sciences, DGIST, Daegu, 42988, Republic of Korea

[6] Graduate Program in Innovative Biomaterials Convergence, Ewha Womans University, Seoul, 03760, Republic of Korea

# These authors equally contributed to this work

*Corresponding authors:

Sun-Shin Cha

Tel: +82-2-3277-3858

Fax: +82-2-3277-2385

E-mail: chajung@ewha.ac.kr

Ji-Sook Hahn

Tel: +82-2-880-9228

Fax: +82-2-888-1604

E-mail: hahnjs@snu.ac.kr

Supplementary methods

Reagents and equipment for proteome analysis

Dithiothreitol (DTT), triethylammonium bicarbonate (TEAB), trifluoroacetic acid (TFA), and iodoacetamide (IAA) were purchased from Sigma-Aldrich (St. Louis, MO, USA). Trypsin was purchased from Promega (Madison, WI, USA). Oasis HLB 1 cc cartridge was purchased from Waters (Milford, MA, USA). Acetonitrile (ACN, cat: 9017-88) and methanol (MeOH, cat: 9093-88) were purchased from Avantor (Radnor, PA, USA). Formic acid (FA) was provided by Wako Pure Chemicals (Osaka, Japan). Dionex UltiMate 3000 RSLC nano System, Q-Exactive Plus, PepMap RSLC C18, Acclaim PepMap 100, HPLC-grade water (cat: W6-4), 0.1% FA in ACN (cat: LS120-212), and 0.1% FA in water (cat: LS118-4) were purchased from Thermo Fisher Scientific (Waltham, MA, USA).

Sample preparation for proteome analysis

Cell lysates of 3 biological replicates from each experimental condition were analyzed in duplicate. 100 µg of cell lysate protein was prepared in 50 mM TEAB buffer and 20 mM DTT final volume up to 20 µL. The protein solution was heated to 95°C for 10 min, and 40 mM of IAA was treated for 30 min in the dark. The protein sample was digested using 1 µg of trypsin for 16 hr at 37°C. To stop tryptic digestion, 1% TFA was added to the solution making a final volume of 800 µL.

Desalting was conducted with an Oasis HLB 1 cc cartridge. A single cartridge was activated by adding 1 mL of MeOH, and equilibrated by rinsing 1 mL of wash buffer (0.1% FA in distilled water). The digested of peptides solution was then applied to the cartridge and washed three times. To elute the peptides, an ACN gradient was utilized. Each 300 µL of elution buffer (20%~80% ACN in 0.1% FA in water, 10% of a step size) was added to the cartridge, and collected in a tube. The eluent was dried using a vacuum centrifuge and stored at –80℃ until further use.

LC–MS/MS analysis of *Methylomonas* DH-1

A sample was dissolved in 20 μL of mobile phase A (0.1% FA and 2% ACN), and 2 μL was injected into an UltiMate 3000 RSLC nano System equipped with PepMap RSLC C18 (2 μm, 100 Å, 75 μm × 50 cm) and Acclaim PepMap 100 (75 μm × 2 cm) columns. Using the mobile phase B (0.1% FA in 80% ACN), separation was performed via following steps: 5 min of isocratic flow in 6% mobile phase B; 6% mobile phase B linear to 50% for 90 min; 50% mobile phase B linear to 95% for 10 min; 5 min of isocratic flow in 95% mobile phase B; 95% mobile phase B linear to 6% for 1 min; 19 min of isocratic flow in 6% mobile phase B. The total run time was 130 min, temperature of the column oven was 35℃, and the flow rate was 300 nL/min.

For MS acquisitions, a Q-Exactive Plus mass spectrometer was utilized. The capillary temperature was set at 275°C and ion spray energy was set to 2.0 kV in positive mode. The auxiliary, sheath, and sweep gas flow rates were set to 0 arbitrary units. In full MS mode, mass scans were obtained at a resolution of 70,000. The automatic gain control (AGC) value was set to 1×10^6, the maximum injection time was 250 ms, and the scan range was 150–2,000 m/z. In MS/MS mode, the data were obtained at a resolution of 17,500 with the top 12 precursor ions excluding 1^+, 7^+, and 8^+ charge states. The AGC value was set to 1×10^5, the maximum injection time was 100 ms, and the isolation window was 2.0 m/z.

Proteome analysis for *Methylomonas* DH-1

To identify proteins from the raw MS data, label-free quantification (LFQ) was performed using MaxQuant (ver1.6.5.0). Based on UniProt reference proteome data (UP000077385), the searching mode was set to trypsin cleavage with a maximum of 2 missed cleavages and all other settings were set as default parameters. The protein groups from LFQ were then arranged via Perseus (ver2.0.6.0). The respective data was filtered by removing potential contaminants, identifications based on sites only, and reverse identification. The LFQ intensities were log2 transformed, and missing

values were replaced according to normal distribution using the imputation function in Perseus. Statistical analysis was performed by two-sided t-test (false discovery rate value for 0.05 and S0 value for 0.1).

Supplementary Tables

Table S1 Strains used in this study

Strain	Description	Genotype	Reference
Methylomonas sp. DH-1	Wild type strain		(1)
JHM80	Evolved strain from DH-1		(2)
JHM102	Evolved strain from JHM80		This study
DH-1 AlaRS^{L219M}	AlaRS L219M mutation in DH-1	DH-1 *Δ(alaRS-aspK)::alaRS*^{L219M}*-aspK-kan*^{R}	This study
JHM80 AlaRS^{L219M}	AlaRS L219M mutation in JHM80	JHM80 *Δ(alaRS-aspK)::alaRS*^{L219M}*-aspK-kan*^{R}	This study
DH-1 AlaRS^{WT}	*kan*^{R} expression in DH-1	DH-1 *Δ(alaRS-aspK)::alaRS-aspK-kan*^{R}	This study

Table S2 Recipe of the stock solutions of NMS

Ingredient	Amount
1000X trace element solution	
$FeSO_4 \cdot 7H_2O$	500 mg/L
$ZnSO_4 \cdot 7H_2O$	400 mg/L
$MnCl_2 \cdot 7H_2O$	20 mg/L
$CoCl_2 \cdot 6H_2O$	50 mg/L
$NiCl_2 \cdot 6H_2O$	10 mg/L
H_3BO_3	15 mg/L
EDTA	250 mg/L
100X vitamin stock	
Biotin	2.0 mg/L
Folic acid	2.0 mg/L
Thiamine HCl	5.0 mg/L
Ca pantothenate	5.0 mg/L
Vitamin B12	0.1 mg/L
Riboflavin	5.0 mg/L
Nicotinamide	5.0 mg/L
100X phosphate stock solution	
KH_2PO_4	26 g/L
Na_2HPO_4	32.8 g/L

Table S3 Plasmids used in this study

Plasmid	Description	Reference
Plasmids for *Methylomonas* sp. DH-1		
pIns	Plasmid containing [U_{ins}-T_{rrnB}-kan^R-D_{ins}] cassette for integration into noncoding region between *AYM39_05845* and *AYM39_05850*	(2)
pIns-alaRS	pInsK-[U_{alaRS}-*alaRS*-*aspK*-T_{rrnB}-kan^R-D_{alaRS}]	This study
pIns-alaRS^{L219M}	pInsK-[U_{alaRS}-$alaRS^{L219M}$-*aspK*-T_{rrnB}-kan^R-D_{alaRS}]	This study
Plasmids for *E. coli*		
pIns-alaRS^{L219F}	pInsK-[U_{alaRS}-$alaRS^{L219F}$-*aspK*-T_{rrnB}-kan^R-D_{alaRS}]	This study
pIns-alaRS^{L219A}	pInsK-[U_{alaRS}-$alaRS^{L219A}$-*aspK*-T_{rrnB}-kan^R-D_{alaRS}]	This study
pET-28b(+)-AlaRS	pET-28b(+)-*alaRS*	This study
pET-28b(+)-AlaRS^{L219M}	pET-28b(+)-$alaRS^{L219M}$	This study
pET-28b(+)-AlaRS^{L219F}	pET-28b(+)-$alaRS^{L219F}$	This study
pET-28b(+)-AlaRS^{L219A}	pET-28b(+)-$alaRS^{L219A}$	This study
pET-28b(+)-AlaRS$^{V204A\ L219M}$	pET-28b(+)-$alaRS^{V204A\ L219M}$	This study
pET-28b(+)-AlaRS$^{V204L\ L219M}$	pET-28b(+)-$alaRS^{V204L\ L219M}$	This study
pET-28b(+)-AlaRS^{V204A}	pET-28b(+)-$alaRS^{V204A}$	This study
pET-28b(+)-AlaRS^{V204L}	pET-28b(+)-$alaRS^{V204L}$	This study
pET-28b(+)-AlaRS$_{429}$	pET-28b(+)-*$alaRS_{429}$	This study
pET-28b(+)-AlaRS$_{429}^{L219M}$	pET-28b(+)-**$alaRS_{429}^{L219M}$	This study

*$alaRS_{429}$, encoding 1-429 of AlaRS

**$alaRS_{429}^{L219M}$, encoding 1-429 of AlaRS^{L219M}

1. Hur, D.H., Na, J.-G. and Lee, E.Y. (2017) Highly efficient bioconversion of methane to methanol using a novel type I Methylomonas sp. DH-1 newly isolated from brewery waste sludge. *Journal of Chemical Technology & Biotechnology*, **92**, 311-318.
2. Lee, J.K., Kim, S., Kim, W., Kim, S., Cha, S., Moon, H., Hur, D.H., Kim, S.-Y., Na, J.-G., Lee, J.W. *et al.* (2019) Efficient production of d-lactate from methane in a lactate-tolerant strain of Methylomonas sp. DH-1 generated by adaptive laboratory evolution. *Biotechnology for Biofuels*, **12**, 234.

Table S4 Primers used in this study

Forward primer	Reverse primer	Usage
Primer sequence for plasmid generation for adopting mutation in *Methylomonas* sp. DH-1		
gcgGCGGCCGCTTTTTGTCGTTGAGAGGGAGCTTAAAAGG	gcgACTAGTctactgctgatccagaccgaATGC	Cloning [U_{alaRS}-*alaRS-aspK*] fragment of pIns-alaRS with *Not*I/*Bcu*I, Cloning [U_{alaRS}-*alaRS*^{L219M}-*aspK*] fragment of pIns-alaRS^{L219M} with *Not*I/*Bcu*I
gcgGGGCCCTCCGCGGTCGTTGAGCGCGA	gcgGAGCTCCGGCAACCATACCGGCACCG	Cloning [D_{alaRS}] fragment of pIns-alaRS, pIns-alaRS^{L219M} with *Apa*I/*Sac*I
Primer sequence for protein purification		
TGACGCCGtTcCCGGCGCCGT	ACGGCGCCGGgAaCGGCGTCA	Adopting L219F mutation on pIns-alaRS
CGCCGgcGCCGGCGCCGTCGG	CCGGCgcCGGCGTCAGGTTGC	Adopting L219A mutation on pIns-alaRS
gcgGGATCCgATGACCAGCGCCGAAATCCGAGCCG	gcgGCGGCCGCACCCAACTTGGCTTTAACCCATACAGGTACCGCAGC	Cloning *alaRS* fragment of pET-28b(+)-AlaRS with *BamH*I/*Not*I, Cloning *alaRS*^{L219M} fragment of pET-28b(+)-AlaRS^{L219M} with *BamH*I/*Not*I, Cloning *alaRS*^{L219F} fragment of pET-28b(+)-AlaRS^{L219F} with *BamH*I/*Not*I, Cloning *alaRS*^{L219A} fragment of pET-28b(+)-AlaRS^{L219A} with *BamH*I/*Not*I
GATCTGGAACCTGGcGTTCATGCAATACG	CGTATTGCATGAACgCCAGGTTCCAGATC	Adopting V204A mutation on pET-28b(+)-AlaRS^{L219M} and pET-28b(+)-AlaRS
ACCTGcTGTTCATGCAATACGAGCG	ATGAACAgCAGGTTCCAGATCTCGA	Adopting V204A mutation on pET-28b(+)-AlaRS^{L219M} and pET-28b(+)-AlaRS
gcgTCTAGAaaggagatataccATGACCAGCGCCGAAATCCG	actCTCGAGTTAGTGATGATGATGATGATGGCCGCCGGCCCGAGCCCGGT	Cloning AlaRS$_{429}$ fragment of pET-28b(+)-AlaRS$_{\mathbf{429}}$ with *Xba*I/*Xho*I, Cloning AlaRS$_{429}^{L219M}$ fragment of pET-28b(+)-AlaRS$_{\mathbf{429}}^{\mathbf{L219M}}$ with *Xba*I/*Xho*I
Primer sequence for tRNA preparation		
taatacgactcactataGGGGCCATAGCTCAGCTGGG	TGGTGGAGCCAGGGAGGATCG	PCR amplification of tRNA^{Ala} sequence in DH-1

Table S5 Data collection and refinement

	**AlaRS$_{429}$ / ATP, Ala**	**AlaRS$_{429}^{L219M}$ / ATP, Ala**
**Data collection**		
PDB code	9JC7	9JDN
Diffraction source	PAL-5C	PAL-5C
Wavelength	0.98	0.98
Temperature (K)	100	100
Rotation range per image (°)	1	1
Total rotation range (°)	360	360
Exposure time per image (s)	0.3	0.2
Space group	$P2_1$	$P2_1$
a, b, c (Å)	78.23, 55.59, 125.72	78.84, 56.57, 125.63
α, β, γ (°)	90.0, 102.1, 90.0	90.0, 102.3, 90.0
Resolution range (Å)	50-1.90	50-2.18
Total no. of reflections	576571	390443
No. of unique reflections	83655	56385
Completeness (%)[a]	99.94(99.86)	99.06(98.44)
Redundancy[a]	6.9(6.2)	6.9(6.8)
I/σ (I)[a]	13.1(1.4)	8.9(1.9)
Rsym (%)[a,b]	10.2(117.0)	13.9(95.6)
**Refinement statistics**		
Resolution range (Å)	29.80-1.90	29.90-2.18
No. of reflections	83640	56385
**No. of atoms**		
Protein	6700	6652
ATP	62	93
Ala	12	12
Water	196	183
**B-factors**		
Protein	30.5	33.8
ATP	33.7	43.8
Ala	42.7	45.6
Water	30.4	32.8
R(R_{free}) (%)[a,c]	19.83(22.98)	20.13(23.36)
**R.m.s. deviations[d]**		
Bond length (Å)	0.008	0.008
Bond angle (°)	0.940	1.020

a. The number in the parentheses is for the outer shell.
b. $R_{sym} = \Sigma_{\Sigma h}\Sigma_{\Sigma I} |I_{h,i}-I_h| / \Sigma_{\Sigma h}\Sigma_{\Sigma I}\Sigma\Sigma I_{h,i}$, where I_h is the mean intensity of the i observations of symmetry-related reflections of h.
c. $R = \Sigma |F_o-F_c| / \Sigma F_o$, where $F_o = F_p$, and F_c is the calculated protein structure factor from the atomic model. R_{free} was calculated with 10% of the reflections.
d. R.m.s. deviations in bond length and angles are the deviations from ideal values.

Supplementary Figures

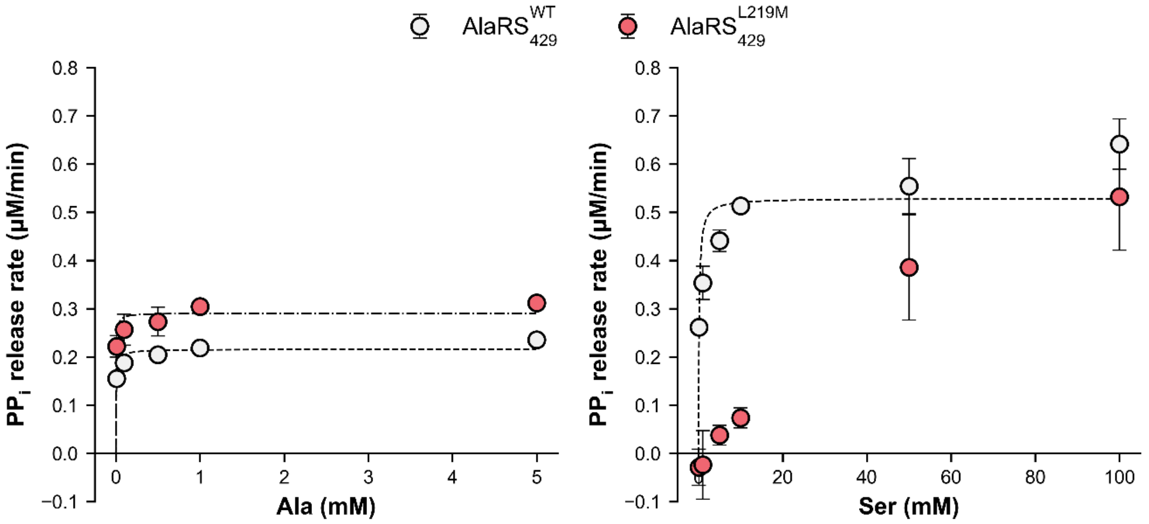

Figure S1. PPi release assay of AlaRS429 and AlaRS429^{L219M} in the presence of tRNA, using Ala and Ser as substrates. Error bars represent s.d. (n = 3)

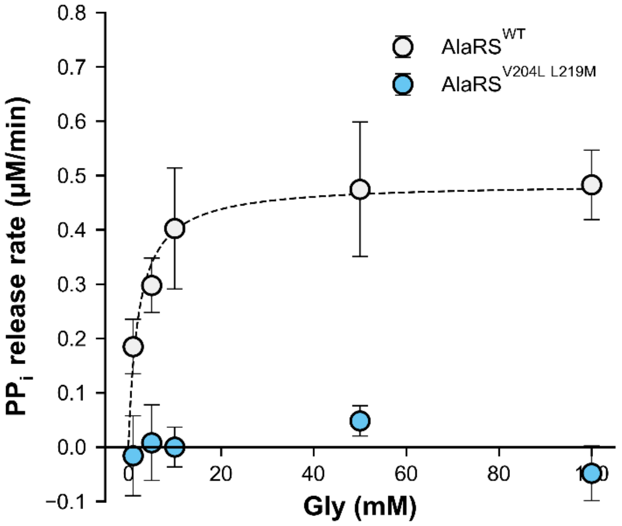

Figure S2. PPi release assay of AlaRS$^{V204L\ L219M}$ in the presence of tRNA using Gly as a substrate. Error bars represent s.d. (n = 3)

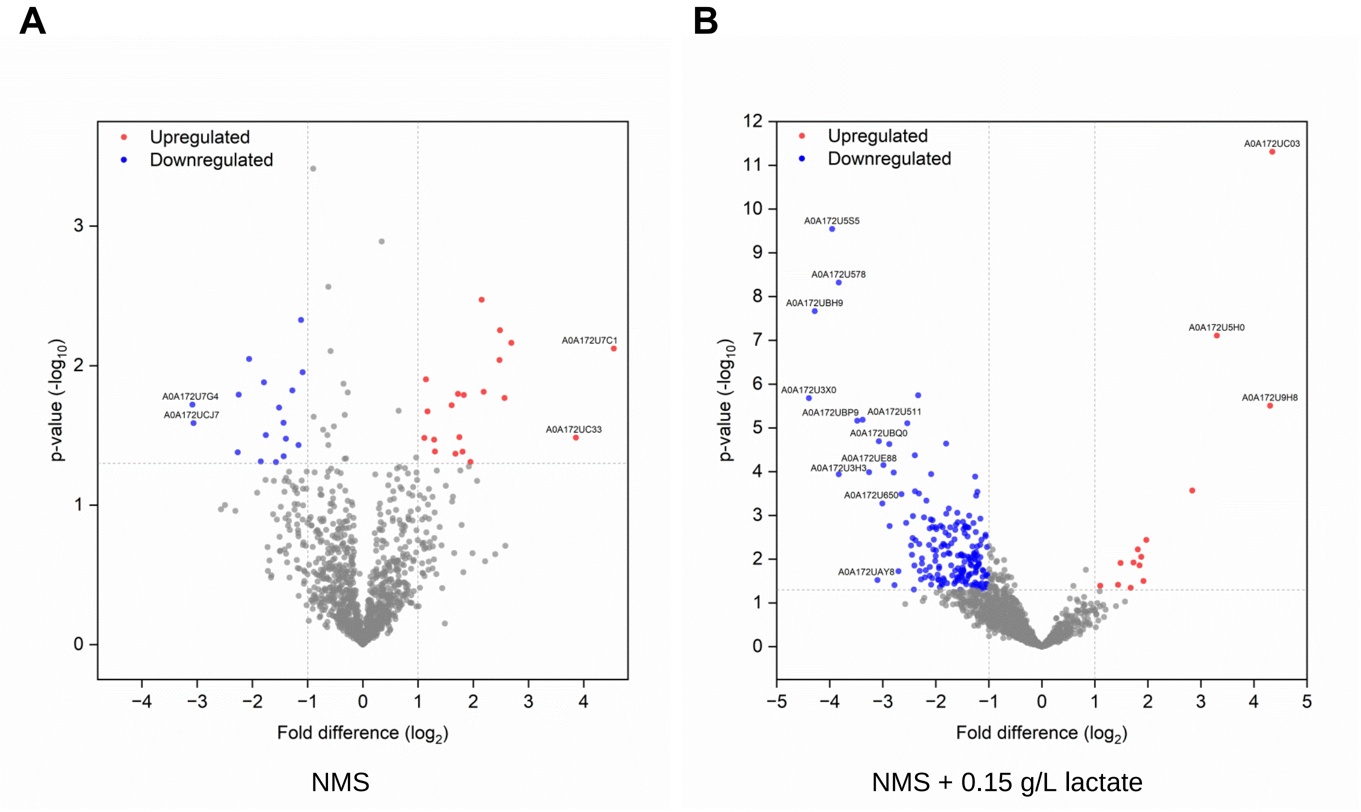

Figure S3. Protein expression patterns of *Methylomonas* sp. DH-1 AlaRS^{WT} and DH-1 AlaRS^{L219M} in NMS media (**A**), and in NMS media containing 0.15 g/L lactate (**B**). 20% (v/v) methane was used as a carbon source. Red dots represent proteins that were expressed at higher levels, while blue dots represent proteins expressed at lower levels in DH-1 AlaRS^{L219M} compared to DH-1 AlaRS^{WT}.
